# Impact of Chronic Prostatitis on the PI-RADS Score 3: Proposal for the Addition of a Novel Binary Suffix

**DOI:** 10.3390/diagnostics11040623

**Published:** 2021-03-30

**Authors:** Sascha Merat, Theresa Blümlein, Markus Klarhöfer, Dominik Nickel, Gad Singer, Frank G. Zöllner, Stefan O. Schoenberg, Rahel A. Kubik-Huch, Daniel Hausmann, Lukas Hefermehl

**Affiliations:** 1 Department of Radiology, Kantonsspital Baden, 5404 Baden, Switzerland; sascha.merat@ksb.ch (S.M.); rahel.kubik@ksb.ch (R.A.K.-H.); 2 Eidgenössische Technische Hochschule (ETH) Zürich, 8092 Zurich, Switzerland; tbluemlein@student.ethz.ch; 3 Siemens Healthcare AG, 8047 Zürich, Switzerland; markus.klarhoefer@siemens-healthineers.com; 4 MR Applications Predevelopment, Siemens Healthcare GmbH, 91052 Erlangen, Germany; marcel.nickel@siemens-healthineers.com; 5 Department of Pathology, Kantonsspital Baden, 5404 Baden, Switzerland; gad.singer@ksb.ch; 6 Computer Assisted Clinical Medicine, Mannheim Institute for Intelligent Systems in Medicine, Medical Faculty Mannheim, Heidelberg University, 68167 Mannheim, Germany; frank.zoellner@medma.uni-heidelberg.de; 7 Department of Radiology and Nuclear Medicine, Medical Faculty Mannheim, University Medical Center Mannheim, Heidelberg University, 68167 Mannheim, Germany; stefan.schoenberg@umm.de; 8 Department of Urology, Kantonsspital Baden, 5404 Baden, Switzerland; lukas.hefermehl@ksb.ch

**Keywords:** prostatitis, PI-RADS, ADC, mpMRI

## Abstract

We examined the impact of chronic prostatitis on the diagnostic performance of multiparametric magnetic resonance imaging (mpMRI). In this retrospective study, 63 men underwent 3T mpMRI followed by MRI/ultrasound fusion biopsy to exclude/confirm clinically significant prostate cancer (csPCa). A total of 93 lesions were included for evaluation. Images were assessed by two radiologists. Prostatitis was graded visually on T2-weighted and contrast-enhanced sequences. The correlation of prostatitis features with the assigned Prostate Imaging Reporting and Data System (PI-RADS) and the presence of csPCa were assessed, and the clinical and functional imaging parameters for differentiating between prostatitis and significant tumors were examined. Histopathological analysis was used as the reference standard. The rate of PI-RADS 3 scores tended to be higher in the presence of radiologically severe prostatitis compared with no/discrete prostatitis (*n* = 52 vs. *n* = 9; *p* = 0.225). In severe prostatitis, csPCa was determined in only 7.7% (4/52) of PI-RADS 3 lesions. In severe chronic prostatitis, a binary prostatitis suffix (e.g., PI-RADS 3 i+ versus i−) within the radiological report may help assess the limitations of mpMRI interpretability because of severe prostatitis and avoid unnecessary biopsies. Mean apparent diffusion coefficient (ADC_mean_) was the best marker (cutoff 0.93 × 10^−3^ mm^2^/s) to differentiate between csPCa/non csPCa in severe prostatitis.

## 1. Introduction

Multiparametric magnetic resonance imaging (mpMRI) is an important clinical tool for the detection and guidance of treatment for prostate cancer (PCa) [1,2,3,4]. Advances in MRI scanner technology and sequence development have allowed the detection of even small PCa lesions with high accuracy. The mpMRI protocol is composed of native T1-weighted (T1W), T2-weighted (T2W), diffusion-weighted imaging (DWI), and dynamic contrast-enhanced (DCE) sequences [5,6]. These sequences are evaluated in a standardized fashion using the Prostate Imaging Reporting and Data System (PI-RADS) v2.1 classification to ensure good diagnostic quality of radiological reports and to increase interobserver agreement by reducing subjectivity in image evaluation [6,7,8]. In cases with abnormal imaging and/or clinical findings (PI-RADS ≥ 3), a prostate biopsy is usually performed. Compared with conventional transrectal ultrasound-guided biopsies, MRI-guided targeted biopsies reduce the incidence of avoidable biopsies and improve the detection rate of clinically significant PCa (csPCa) [3].

Chronic prostatitis is a very common disease in older people and usually progresses without symptoms. Fluctuating and slightly elevated prostate-specific antigen (PSA) levels are often detected by the urologist or general practitioner, which necessitates further work-up with MRI to rule out PCa [6,9]. In imaging, prostatitis is characterized by streaky to wedge-shaped decreases in T2W signals along the prostatic septa of the peripheral zone (PZ) with increased contrast enhancement, a discrete DWI signal increase, and a low to moderate decrease in the apparent diffusion coefficient (ADC). However, in more pronounced findings, the changes may also be diffuse or patchy and indistinguishable from a significant tumor [10]. In these cases, diffuse contrast enhancement in extensive prostatitis limits the use of DCE as a decisive parameter in distinguishing PI-RADS 3 from PI-RADS 4 lesions in the peripheral zone because focal enhancement of lesions can be masked. The degree of the reduction in ADC can help to discriminate between csPCa with a lower ADC and prostatitis with a higher ADC, although there is a significant overlap [11]. Furthermore, because of differences in manufacturers, MRI scanners, and sequences, there are no established ADC thresholds for differentiating between PCa and prostatitis.

In PI-RADS v2, the presence of prostatitis may reduce diagnostic accuracy [12]. Nevertheless, despite the updates of PI-RADS v2 in 2015 and v2.1 in 2019, the fact that prostatitis leads to more difficult assessment of mpMRI remains largely unappreciated [10,12,13]. For example, Rourke et al. reported that inflammation can mimic PCa [14], while Liddell et al. specifically examined PI-RADS 3 lesions and found no csPCa [15]. Other studies have also reported the diagnostic performance of quantitative parameters obtained from mpMRI for differentiating PCa from prostatitis [16,17]. Herein, we hypothesized that severe prostatitis causes an increased number of assigned PI-RADS 3 lesions in which biopsies show no tumors or a clinically insignificant tumor, resulting in a high number of men undergoing unnecessary biopsies. Furthermore, we assessed the efficacy of different clinical and functional DWI and DCE parameters for improving the differentiation between prostatitis and csPCa.

## 2. Materials and Methods

### 2.1. Patients and Lesions

**Figure 1:**
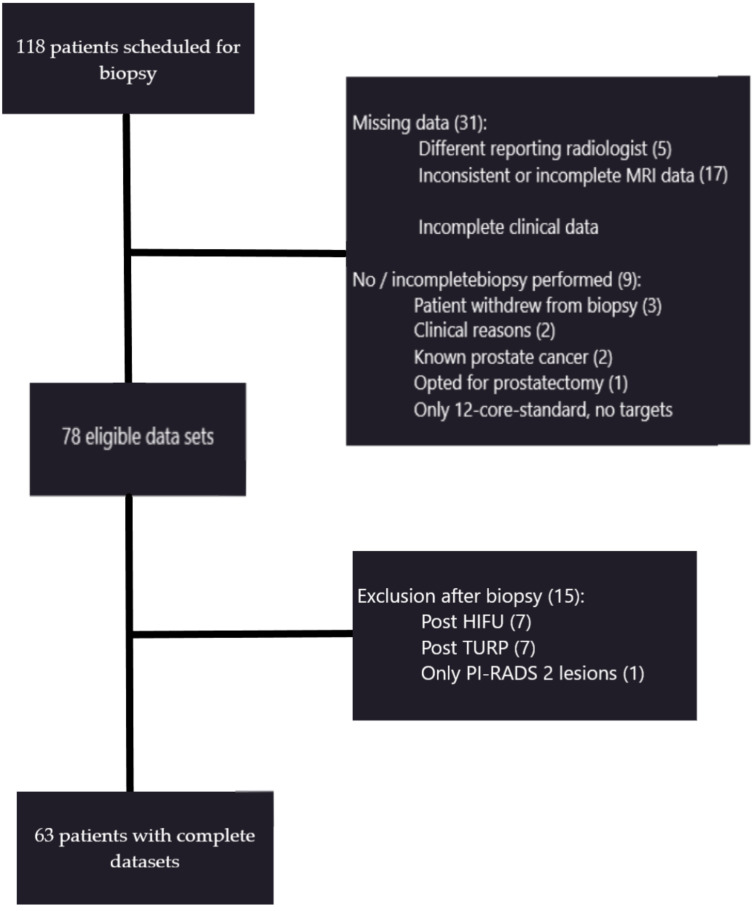
Our patient cohort and exclusion criteria compiled according to individual reasons. Of the initial 118 patients, 63 patients were included in the final evaluation. PI-RADS, Prostate Imaging Reporting and Data System; HIFU, High-intensity focused Ultrasound; TURP, Transurethral resection of the prostate.

Between April 2019 and December 2019, 63 patients (64.3 ± 5.7 years) who received a clinically indicated mpMRI of the prostate followed by an MRI/ultrasound (US) fusion biopsy to rule out or confirm PCa were included in this retrospective study. The mean PSA, prostate volume, and mean PSA density were 9.69 (1.03–139.00) µg/L, 66.2 (20.6–182.0) mL, and 0.165 (0.015–2.000) ng/mL^2^, respectively. The predefined exclusion criteria are detailed in Figure 1. After exclusions, this cohort consisted of 63 patients and 142 lesions, of which 93 lesions were located in the PZ and included for evaluation.

### 2.2. MRI

All examinations were performed with a clinical 3T MRI scanner (MAGNETOM Skyra Fit, Siemens Healthcare, Erlangen, Germany) equipped with a 50-channel coil setup (18-channel body coil and 32-channel spine coil). Patients were positioned head-first supine in the MRI machine. The MRI protocol was designed according to the recommendations of the PI-RADS v2.1 guidelines and consisted of high-resolution T2W Turbo Spin Echo sequences (axial/sagittal/coronal planes; slice thickness, 3 mm; field of view (FOV), 180 mm; repetition time (TR), 4000 ms; echo time (TE), 112 ms), DWI using the RESOLVE sequence (b = 50/800 s/mm^2^; slice thickness, 3 mm; FOV, 170 mm; TR, 4900 ms; TE, 58 ms) with a calculated ultrahigh b-value (b = 2000 s/mm^2^) and a parametric ADC map, and a native T1 and prototypical dynamic T1 gradient echo sequence (csVIBE) for DCE with high temporal resolution (temporal resolution, 10 s; slice thickness, 3 mm; FOV, 260 mm; TR, 3.98 ms; TE, 1.46 ms, flip angle 12°). The csVIBE was acquired during free breathing after administration of 0.1 mmol/kg gadopentetate dimeglumine (Dotarem®; Guerbet, Paris, France) at a rate of 2 mL/s and subsequently reconstructed by compressed sensing. 

### 2.3. Prostatitis Assessment

**Figure 2:**
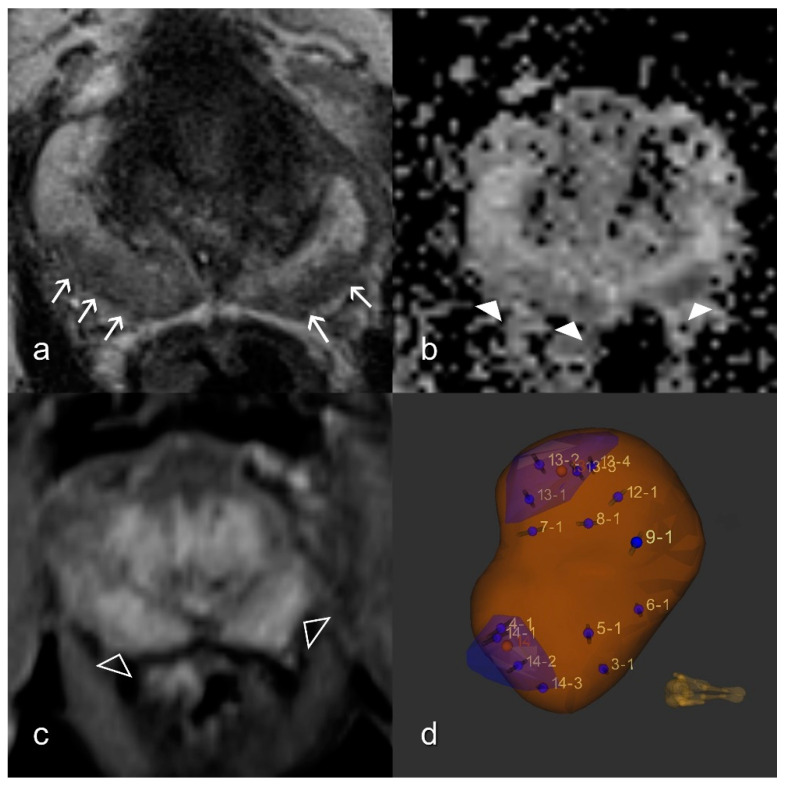
Reference standard. Imaging findings showing patchy T2W signal attenuation (white arrows) in the peripheral zone on both sides (**a**), moderate reduction (white arrowheads) in the ADC map (**b**), and diffuse enhancement (empty arrowheads) in the dynamic T1W VIBE (**c**); Formally, this represents the PI-RADS 3 constellation; (**d**) The MRI/ultrasound (US)-guided biopsy 3D model depicts the target lesions in the blue zones and the template biopsy cores (numbered). Pathology confirmed no malignancy. ADC, apparent diffusion coefficient; T1W, T1-weighted; T2W, T2-weighted; VIBE, Volumetric interpolated breath-hold examination.

According to the PI-RADS v2 guidelines, prostatitis in the PZ results in wedge-shaped or linear or diffuse rather than focal, round, oval, or irregular decreased signals on T2W, and perfusion may also be increased [12]. The gold standard was the presence of the morphologic criteria of prostatitis as defined by PI-RADS v2 and the histopathologic absence of significant tumor in the MR-guided biopsies. A systematic histopathological evaluation of prostatitis was not performed (Figure 2). 

**Figure 3:**
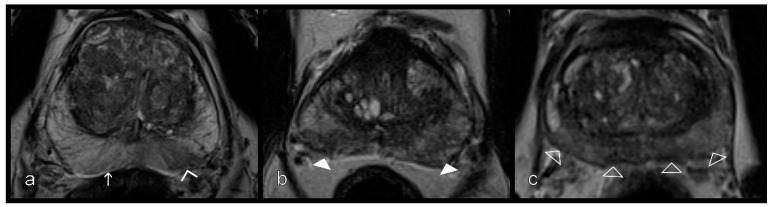
Inflammatory changes in the peripheral zone from left to right. (**a**) Stripes (white arrow) and sharp wedges (empty arrowheads), (**b**) wedges with obscured margins (white arrowheads), and (**c**) patchy and diffuse signal reduction (empty arrowheads) on T2W sequences.

**Figure 4:**
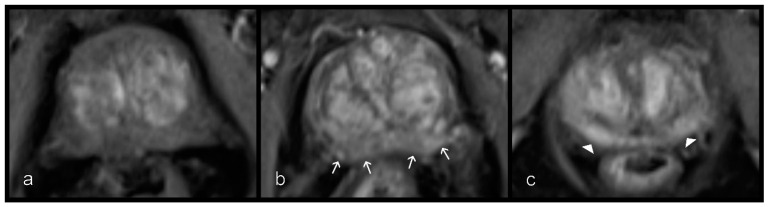
Different levels of enhancement on contrast-enhanced csVIBE sequences in the peripheral zone. (**a**) No, (**b**) mild (white arrows), and (**c**) severe and early enhancement (arrowheads). No and mild enhancement were combined. csVIBE, native T1 and prototypical dynamic T1 gradient echo sequence.

Two readers who were radiologists with 12 years (D.H.) and 5 years (S.M.) of experience in prostate MRI graded the prostatitis independently using the axial T2W and the DCE according to the scheme shown in Figure 3 and Figure 4. On T2W, a distinction was made between the striped and wedge-shaped signal reductions with sharp margins from the extensive, blurred, wedge-shaped, patchy, and diffuse signal attenuation. Because of the small group size of this patient-based evaluation, for statistical analysis, the striped and wedge-shaped signal reductions with sharp margins were summarized as ‘mild’, while the other groups were summarized as ‘severe’ (Table 1). The optimal contrast phase was selected from the multiphase csVIBE (usually approximately 80 s after the sequence was started), and the enhancement pattern was rated (0 = no enhancement, 1 = mild enhancement, 2 = severe enhancement). As above, the no- and mild-enhancement ratings were combined for statistical analysis (Table 1). 

**Figure 5:**
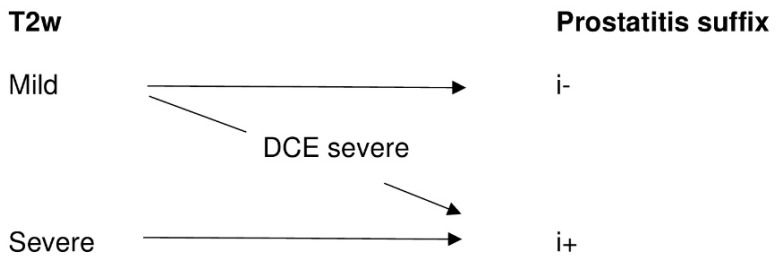
The algorithm used for our proposed inflammation suffix. Mild T2W changes (as described in Table 1) are classified as ‘i−’ but can be upgraded to ‘i+’ when severe contrast enhancement is present. Severe T2W changes are classified as ‘i+’ regardless of the enhancement pattern.

From this assessment, we derived an algorithm for a simple inflammation suffix, as follows: mild findings in T2W were labeled as ‘i−’ (no inflammation) but were upgraded to ‘i+’ (inflammation) if DCE showed severe enhancement, while severe findings in T2W were labeled as ‘i+’ regardless of DCE (Figure 5). We assumed that an increased enhancement indicated the inflammatory activity of chronic prostatitis.

### 2.4. DWI 

The minimum, maximum, and mean ADC values were measured in each lesion with a PI-RADS score ≥3. The measured area was kept constant (11.5 mm^2^ circle shape) and was only altered if tissue obviously located outside of the lesion would have been included. 

### 2.5. DCE Analysis

The csVIBE sequence was used to analyze the contrast kinetics. We applied the two-compartment Tofts model, where a systemically administered contrast agent penetrates a capillary wall of increased permeability into the interstitium. The amount of contrast accumulation in the interstitium is dependent on capillary permeability, the difference in the concentration on either side of the capillary wall, and on the total surface area [18]. In neoplastic and inflammatory processes, the capillary permeability is increased [19]. Pharmacokinetic analysis was performed with syngo.via (Tissue4D version VB30; Siemens Healthcare, Erlangen, Germany). Regions of interest (ROIs) of similar size were precisely placed in each lesion to measure K_trans_ (volume transfer rate reflecting the efflux rate of gadolinium contrast from blood plasma into the tissue extravascular extracellular space (EES), K_ep_ (time constant for gadolinium contrast reflux from EES back into blood plasma), V_e_ (fractional volume of the EES), area under the curve (AUC), and Chi-square values [18], as well as for evaluating the kinetic curve type (Type 1, continuous wash-in; Type 2, early wash-in followed by plateau phase; Type 3, early wash-in followed by wash-out) [19].

### 2.6. MR/US Fusion-Guided Biopsy and Gold Standard

The same experienced radiologist (12 years of experience) reported all prostate MRI examinations and subsequently performed the lesion segmentation (Profuse software v3.1.8.4; Eigen, Canada). MRI conventional transrectal ultrasound-guided biopsies were performed by urology consultants with a median experience of 5 years using an Artemis device (Artemis; Eigen, CA, USA) according to the European Association of Urology guidelines [20].

Depending on the lesion sizes, between 2 and 4 cores of each target lesion and complementary template biopsies of the remainder of the prostate (5–12 cores depending on the prostate volume already covered by targeted biopsies) were sampled. This lesion-based histopathological evaluation served as the gold standard for our examination. Clinically significant cancer was defined as a Gleason score ≥4 + 3 (Grade 7b and higher) or a maximum cancer core length of ≥6 mm according to the Epstein criteria [21]. Only biopsies from the PZ with PI-RADS scores of 3–5 were considered for evaluation, as prostatitis in the transitional zone is barely visible because of the physiologically heterogeneous appearance of this glandular region.

### 2.7. Statistical Analyses

Only the ratings of the experienced radiologist were used for the statistical evaluation. The ratings of the less experienced radiologist were used to determine the interobserver agreement using Cohen’s kappa. The Chi-square test was used to evaluate the correlation between PI-RADS scores and prostatitis. Here, the PI-RADS 4 and 5 lesions were combined, and no corrections were used. An approximately normal distribution was verified in pair plots with bell-shaped histograms. Where this was not the case, the logarithm was used (e.g., for PSA-dependent variables). Univariate two-sample *t*-tests were used to determine variables with discriminating power for the different clinical and functional imaging parameters (ADC_mean_, ADC_min_, ADC_max_, K_ep_, K_trans_, PSA_velocity_, log(PSA_density_), log(PSA_latest_)) and were judged by their respective *p*-values. Bonferroni correction was applied to account for multiple testing (adjusted *p*-values; p_adj_ = *p* × *n* (*n* = 8)). A *p*-value of <0.05 was considered statistically significant.

Using logistic regression, the area under the receiver operating characteristic (ROC) curve for univariate observation of ADC_mean_ for the differentiation between csPCa and non-csPCa/no tumor was used for no/discrete prostatitis and severe prostatitis. Sensitivity, specificity, and corresponding cutoffs were calculated. All statistical analyses were performed with statistical software (Python (www.python.org), using sklearn, numpy, and matplotlib libraries for ROC analysis) (R; R Foundation for Statistical Computing, Vienna, Austria; using ggplot2, pROC, and GGally libraries for descriptive statistics, tests, and graphics).

## 3. Results

### 3.1. Lesions and Histopathologic Findings 

Among the 93 lesions in the PZ, csPCa was histopathologically confirmed in the targeted biopsy in 18 lesions. Insignificant cancer was found in 12 target lesions. The 63 remaining targeted biopsies were negative. On the template biopsy, only one additional csPCa was detected, although no significant tumor was found in the target biopsy.

### 3.2. Relationship Between PI-RADS Score, csPCa, and Prostatitis

Chronic prostatitis was rated as severe in 53 patients (severe without enhancement, *n* = 31; severe with enhancement, *n* = 22) and as not evident/discrete in 11 patients. Cohen’s kappa for inter-reader agreement was substantial for both groups (0.78 for T2W grading; 0.68 for contrast enhancement). Overall, 65.6% of lesions (61/93) were classified as PI-RADS 3, 25.8% (24/93) as PI-RADS 4, and 8.6% (8/93) as PI-RADS 5. 

**Figure 6:**
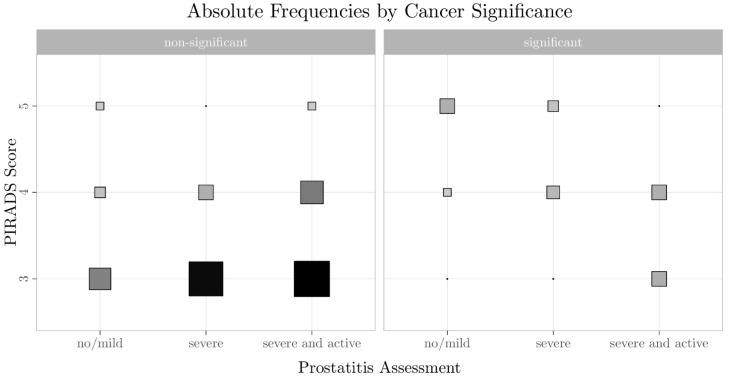
The relationship between the PI-RADS scores, the presence of clinically significant prostate cancer (csPCa)/non-csPCa, and the prostatitis rating in the peripheral zone (PZ). Particularly in diffuse inflammatory active prostatitis, PI-RADS 3 scores corresponded to non-csPCa in the majority of cases. The underlying data are shown in Table 3.

There was a trend towards a PI-RADS score of 3 being assigned more frequently with severe prostatitis (PI-RADS 3, 68.4%; PI-RADS 4, 27.6%; PI-RADS 5, 3.9%) compared with no/discrete prostatitis (PI-RADS 3, 52.9%; PI-RADS 4, 17.6%; PI-RADS 5, 29.4%; *p* = 0.225), especially if there was also an increased contrast enhancement as an indicator of inflammatory activity (Figure 6). With the current sample size, the test power was 23%, while a sample size of 819 lesions was necessary to achieve a power of 95%. In severe prostatitis, csPCa was determined in only 7.7% (4/52) of PI-RADS 3 lesions (Table 2 and Table 3).

### 3.3. Evaluation of the Performance of Clinical and Functional Imaging Parameters to Predict csPCa

**Figure 7:**
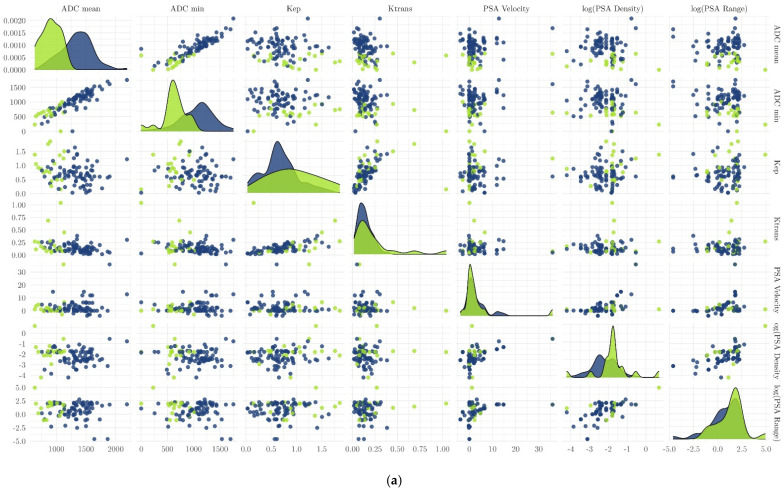

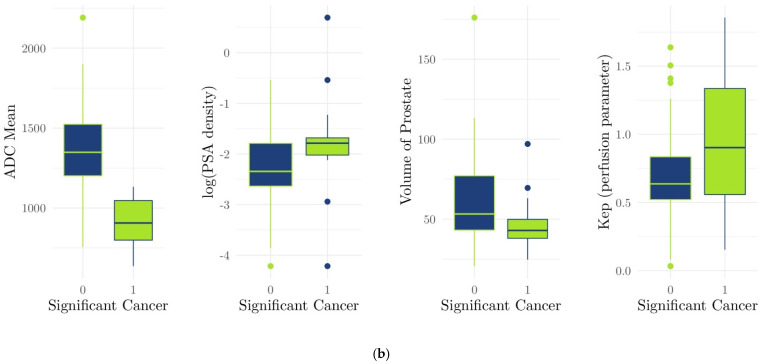
(**a**) and (**b**) Performance of clinical and imaging parameters to discriminate between csPCa and non-csPCa. Of all clinical and functional imaging parameters, the diffusion parameters ADC_mean/min_ had the best discrimination ability for the presence of a significant (green) or non-significant tumor/no tumor (blue) in the peripheral zone. Adjusted *p*-values for each variable are as follows: p(ADC_mean_) = <0.001, p(log(PSA_density_)) = 0.428, p(VolumeProstate) = 0.024, p(K_ep_) = 0.249.

**Figure 8:**
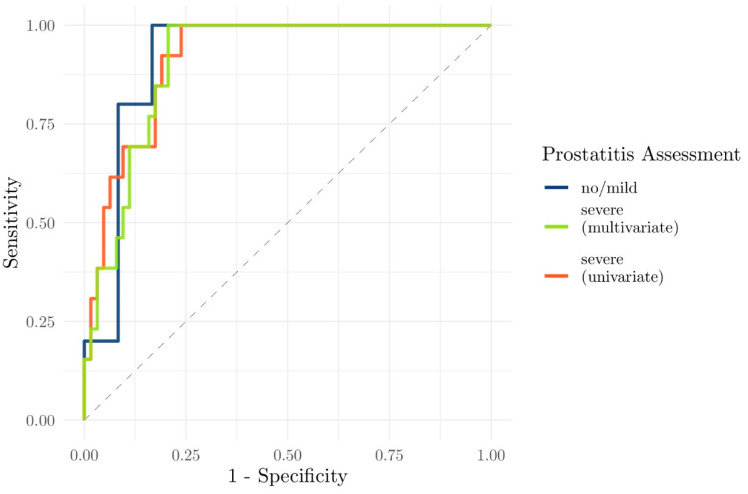
Univariate (ADC_mean_) and multivariate (ADC_mean_, K_ep_, and PSA_density_) receiver operating characteristic (ROC) analysis of clinical and imaging parameters to differentiate csPCa/non-csPCa showing moderate sensitivity/specificity. The univariate (ADC_mean_ = blue) curves for no/mild prostatitis and severe prostatitis were identical, so only one curve was plotted.

ADC values (particularly the ADC_mean_) were the best discriminators between csPCa and non-significant PCa/no tumor (Figure 7a,b; Table 4). Using logistic regression, the area under the ROC curve for univariate observation of ADC_mean_ for the differentiation between csPCa and non-csPCa/no tumor was 0.92 (95% confidence interval (CI), 0.76–1.00) in no/discrete prostatitis and 0.92 (95% CI, 0.85–0.98) in severe prostatitis. The AUCs were not improved using multivariate ROC curves (ADC_mean_, K_ep_, and PSA density combined, 0.92 (95% CI, 0.76–1.00) in no/discrete prostatitis and 0.91 (95% CI, 0.84–0.97) in severe prostatitis) (Figure 8). 

In the univariate assessment (ADC_mean_), the sensitivity for csPCa was 1 for either no/mild prostatitis and severe prostatitis, with a specificity of 0.83 and 0.76, respectively. The ADC_mean_ cutoff for differentiating between csPCa/non-csPCa was 0.9282 × 10^−3^ mm^2^/s for no/mild prostatitis and 0.9285 × 10^−3^ mm^2^/s for severe prostatitis. In the multivariate assessment (ADC_mean_, K_ep_, and PSA_density_), the sensitivity for csPCa was also 1 for either no/mild prostatitis and severe prostatitis, with a specificity of 0.83 and 0.79, respectively. Note that because of the small sample size (e.g., only five lesions with csPCa in the setting of no/mild prostatitis), there was insufficient power for this evaluation.

## 4. Discussion

The present study showed that a PI-RADS 3 score was frequently assigned in the setting of a severe and inflammatory active prostatitis, as defined by the presence of MRI features (severe prostatitis, PI-RADS 3, 68.4%; no/discrete prostatitis, PI-RADS 3, 52.9%), while csPCa was rarely detected histopathologically in these PI-RADS 3 lesions (7.7% (4/52); Table 3). We suggest that this results in numerous unnecessary biopsies or re-biopsies because of the uncertainty of the referring physicians and patients. To our knowledge, this is the first report of a prostatitis score for implementation in PI-RADS to address the limited diagnostic confidence associated with severe prostatitis. By contrast, we found that PI-RADS 4 and 5 lesions corresponded to a large proportion of histopathologically proven significant tumors. Thus, a prostatitis score is not necessary in these cases, as these patients have to be biopsied anyway.

In the current version of the PI-RADS classification system (PI-RADS v2.1) [13], DWI is considered the dominant sequence for assessing the PZ, while high-resolution T2W is predominantly used for assessing the transitional zone. DCE is only of minor importance and contributes alone to the decision between PI-RADS 3 (the presence of clinically significant cancer is equivocal) and PI-RADS 4 (clinically significant cancer is likely to be present) lesions in the PZ. Zhou et al. showed significantly lower ADC in tumours than in normal tissue [22], and Assinder et al. acknowledged that numerous other studies proved DWI to be the best predictor of prostate cancer lesions in mpMRI [23]. Our finding that DWI, and in particular ADC, is the best available clinical and imaging marker for differentiating between csPCa and prostatitis in the PZ is consistent with previous reports (Figure 7a,b). However, an overlap of ADC values between PCa and prostatitis is often described [11,24]. For example, Nagel et al. performed a study with 88 eligible patients (116 biopsy specimens) who underwent mpMRI of the prostate at 3T, with reported median ADC values in normal tissue, prostatitis, low-grade (Gleason score components 2 or 3) PCa, and high-grade (Gleason score components 4 or 5) PCa of 1.22 ± 0.21 × 10^−3^ mm^2^/s, 1.08 ± 0.18 × 10^−3^ mm^2^/s, 0.88 ± 0.15 × 10^−3^ mm^2^/s, and 0.88 ± 0.13 × 10^−3^ mm^2^/s, respectively. In that study, there were significant differences between prostatitis and both high- and low-grade PCa (*p* < 0.001), although there was a clear overlap of ADC values [11]. In another study analyzing 85 suspicious lesions and their correlation with histopathology, 44 lesions showed PCa (51.8%), 21 chronic prostatitis (24.7%), and 20 others benign tissue (23.5%). Furthermore, prostatitis usually had borderline PI-RADS scores, while an ADC ≥ 0.9 × 10^−3^ mm^2^/s achieved the highest predictive value for prostatitis (AUC, 0.859) [24]. This is consistent with our results with an ADC cutoff of 0.93 × 10^−3^ mm^2^/s for differentiating between csPCa and non-csPCa.

Several studies have examined whether prostatitis and PCa can be discriminated with PI-RADS. A study by Zhang et al. of 77 patients with PCa and 29 with prostatitis showed that prostatitis and csPCa of the PZ could be distinguished by PI-RADS v1 and v2 [25]. Liddell et al. specifically examined PI-RADS 3 lesions and found no csPCa; in that study, six of 92 lesions with PCa were Gleason 7a (3 + 4) or lower [15]. Jordan et al. also assessed the diagnostic accuracy of ADC values in combination with PI-RADS v2 for diagnosis of csPCa compared with PI-RADS v2 alone, with the main benefit of ADC being an improved differentiation of csPCa/non-csPCa in PI-RADS 4 lesions [26]. Furthermore, prostate volume and the ratio of ADC tumors in the contralateral prostate were shown to predict csPCa in PI-RADS 3 lesions, albeit with a rather low sensitivity of 59% and specificity of 88% [27].

Interestingly, Rourke et al. showed that inflammation can mimic PCa, with almost 45.9% of biopsies being negative for malignancy, although the PI-RADS score for each lesion was not assigned [14]. In contrast to our findings, Uysal et al. found that ADC, quantitative pharmacokinetic parameters (K_trans_, k_ep_, V_e_, and V_p_), the lesion-to-normal prostate tissue ratios, and time-to-peak (TTP) values could successfully differentiate between PCa and prostatitis [17]. Furthermore, Sureka et al. reported good differentiation using the quantitative contrast-enhancement technique, although with a very small sample size (*n* = 32) [16].

It remains controversial whether quantitative DCE-MRI parameters improve the diagnostic performance of mpMRI for distinguishing between normal tissue and PCa, including differentiation of cancer grades [19]. Furthermore, it was previously suggested that PSA density could replace DCE altogether [28]. Nevertheless, an advantage of the multiphase csVIBE used for DCE was the simultaneous high morphological resolution, which allows detailed assessment of the contrast behavior of prostatitis for our prostatitis score. This technique also allows the patient to breathe freely without compromising image quality [29], and perfusion maps can be generated with advanced motion correction. In the present study, we found no significant benefit of the calculated perfusion parameters according to a Tofts model for differentiating between prostatitis and csPCa (K_ep_, adj. *p* = 0.32; K_trans_, adj. *p* = 1; Table 3). 

We also found that the biochemical parameters (PSA_velocity_, adj. *p* = 1; log(PSA_density_), adj. *p* = 0.38; log(PSA_latest_), adj. *p* = 1) did not show a significant benefit for discrimination between csPCa and non-csPCa. Polanec et al. investigated the potential to integrate clinical and biochemical patient parameters to avoid unnecessary MRI-guided biopsies. In that study, patient characteristics (age, prostate volume), biochemical parameters (PSA levels, PSA density), and PI-RADS v2 scores were evaluated, with the combined regression model (age, PSA density, PI-RADS v2 score) providing an AUC of 0.84 to predict csPCa (significantly superior to each single parameter; *p* ≤ 0.0009). Furthermore, biopsies could have been avoided in 50% of patients (64/128), while csPCa would have been missed in only 4% (2/50) [30]. In the present study, the combination of clinical and imaging parameters did not significantly improve the AUC compared with ADC alone. In our cohort, the performance of ADC was very robust regardless of the degree of prostatitis (0.92 (95% CI, 0.76–1.00) in no/discrete prostatitis; 0.92 (95% CI, 0.85–0.98) in severe prostatitis). If this should prove to be consistent with larger cohorts, it may become a useful parameter to help decide whether a biopsy should be performed or not.

Our study has some major limitations. First, the prostatitis grading was performed visually based on the observation of T2W and DCE, while histopathological evidence was not obtained. Long-term PSA levels were not evaluated. Furthermore, our cohort was too small to detect statistically significant differences between the groups for the association between prostatitis, csPCa/non-csPCa, and PI-RADS 3 scores. In this context, the groups with PI-RADS scores of 4 and 5 and only low/no prostatitis were very small.

## 5. Conclusions

We found that PI-RADS 3 lesions of the PZ often correspond to prostatitis and that after MRI/US-guided biopsy, these areas rarely show a csPCa. We believe that this is a diagnostic issue leading to significant limitations in the interpretability of mpMRI, which has not been sufficiently appreciated in PI-RADS to date. To address this problem, we recommend that a prostatitis suffix be implemented in the radiological report for PI-RADS 3 findings in the PZ. We suggest a binary nomenclature for inflammation with the suffix i+ and i− (e.g., PI-RADS 3 with significant inflammation = PI-RADS 3 i+; PI-RADS 3 without significant inflammation = PI-RADS 3 i−; Figure 5). Concerning patient management, PI-RADS 3i+ lesions with fluctuating or stable PSA and initial negative biopsy might not require rebiopsy. Hence, omitting rebiopsy in such cases could potentially avoid bothersome biopsy-related symptoms and lead to a more cost-effective management.

Furthermore, in the PZ, ADC was the best marker in our small cohort to reliably differentiate between csPCa and benign changes or non-significant tumors, regardless of the degree of prostatitis. This underlines the importance of establishing an ADC threshold to avoid unnecessary PI-RADS 3 scores and biopsies and to improve interobserver agreement and diagnostic confidence.

## Figures and Tables

**Table 1:** On T2W, striped signal attenuations and sharp wedges were defined as mild, while blurry wedge-shaped, patchy, and diffuse alterations were combined and defined as severe. For dynamic contrast-enhanced (DCE) imaging, no or mild enhancement were combined.

Prostatitis Grading	Mild	Severe
T2W	Stripes/sharp wedges	Wedges with obscured margins Patchy Diffuse
	**No/mild**	**Severe**
DCE	No Mild	Severe and early

**Table 2:** Absolute values for the association between the PI-RADS scores and prostatitis grade in the peripheral zone with totals, combining severe prostatitis with and without positive DCE, and disregarding the histopathological results of prostate cancer.

PI-RADS Score	No/Discrete Prostatitis	Severe Prostatitis	Total
PI-RADS 5	5	3	8
PI-RADS 4	3	21	24
PI-RADS 3	9	52	61
Total	17	76	93

**Table 3:** Absolute values for the association between PI-RADS scores, prostatitis grade, and non-csPCa/PCa in the peripheral zone. The combination of PI-RADS 3 and severe or active chronic prostatitis was the most common, while clinically significant prostate cancer (csPCa) was rarely detected in this combination. Note that the group sizes for PI-RADS 4 and 5 and no/discrete prostatitis were very small.

PI-RADS Score	Non-Significant Cancer			Significant Cancer		
	No/Discrete Prostatitis	Severe Prostatitis	Severe/Active Prostatitis	No/Discrete Prostatitis	Severe Prostatitis	Severe/Active Prostatitis
PI-RADS 5	1	0	1	4	2	0
PI-RADS 4	2	4	10	1	3	4
PI-RADS 3	3	23	25	0	0	4

**Table 4:** Clinical and imaging parameters to discriminate csPCa and non-csPCa/no tumor.

Variable	Mean (SD)	*t*-Statistic	*p*-Value	Adj. *p*-Value
ADC_min_	985.3 (354.7)	6.639	<0.001	<0.001
ADC_mean_	1272.1 (309.0)	9.346	<0.001	<0.001
ADC_max_	1539.4 (3448)	7.205	<0.001	<0.001
K_ep_	0.726 (0.388)	−2.309	0.031	0.249
K_trans_	0.145 (0.099)	−1.445	0.166	1
PSA_velocity_	2.778 (6.282)	−0.302	0.766	1
log(PSA_density_)	−2.159 (0.769)	−2.063	0.053	0.428
log(PSA_latest_)	1.878 (0.669)	−1.736	0.096	0.768

## Data Availability

A copy of the raw data set has been uploaded upon submission.
